# Striatal dopaminergic neurons as a potential target for GDNF based ischemic stroke therapy

**DOI:** 10.3906/sag-2108-268

**Published:** 2021-11-11

**Authors:** Mustafa Çağlar BEKER, Merve BEKER, Ahmet Burak ÇAĞLAYAN, Busenur BOLAT, Ülkan KILIÇ, Gamze TORUN KÖSE, Ertuğrul KILIÇ

**Affiliations:** 1 Department of Physiology, School of Medicine, Istanbul Medipol University, İstanbul, Turkey; 2 Regenerative and Restorative Medicine Research Center (REMER), Research Institute for Health Sciences and Technologies (SABITA), İstanbul Medipol University, İstanbul, Turkey; 3 Department of Medical Biology, Hamidiye International School of Medicine, University of Health Sciences Turkey, İstanbul, Turkey; 4 Department of Medical Biology, Institute of Health Sciences, University of Health Sciences Turkey, İstanbul, Turkey; 5 Department of Medical Biology, Hamidiye School of Medicine, University of Health Sciences Turkey, İstanbul, Turkey; 6 Department of Genetics and Bioengineering, Faculty of Engineering, Yeditepe University, İstanbul, Turkey

**Keywords:** Dopaminergic neurons, GDNF, gene therapy, ischemia, lentivirus

## Abstract

**Background/aim:**

Glial cell-line-derived neurotrophic factor (GDNF) is a well-known regulatory neurotrophic factor on dopaminergic neurons. Several pathologies have been documented so far in case of any impairment in the dopaminergic system. This study aimed to investigate the potential protective role of lentiviral GNDF delivery on the small population of tyrosine hydroxylase (TH) positive dopamine producing striatal neurons after ischemic stroke.

**Materials and methods:**

Fourteen C57BL/6J male mice (8–10 weeks) were intracerebrally treated with lentiviral GDNF (Lv-GDNF) or vehicle. Ten days after injections, cerebral ischemia was induced by blockage of the middle cerebral artery. Animals were terminated 72 h after ischemia, and their brains were taken for histological and molecular investigations. Following confirmation of GDNF overexpression, TH immunostaining and immunoblotting were used to evaluate the role of GDNF on dopaminergic neurons. Next, Fluro Jade C staining was implemented to examine the degree of neuronal degeneration at the damaged parenchyma.

**Results:**

Neither the amount of TH positive dopaminergic neurons nor the expression of TH changed in the Lv-GDNF treated animals comparing to the vehicle group. On the other hand, GDNF exposure caused a significant increase in the expression of Nurr1, an essential transcription factor for dopaminergic neurons and Gap43, growth and plasticity promoting protein, in the ischemic striatum. Treatment with Lv-GDNF gave rise to a significant reduction in the number of degenerated neurons. Finally, enhanced GDNF expression also induced expression of an important stress-related transcription factor NF-κB as well as the nitric oxide synthase enzymes iNOS and nNOS in the contralesional hemisphere.

**Conclusion:**

Considering these results together, GDNF’s impact on the survival of striatal dopaminergic neurons is not outstanding for its neuroprotective role. However, it seems that GDNF conducts several signaling pathways by acting on key transcription factors and shows its protective feature by fine-tuning the degeneration-related processes.

## 1. Introduction

Stroke is a leading cause of death and long-term disabilities worldwide. Current treatment approaches for stroke are restricted to fibrinolytic therapies within a narrow time window after the ischemic attack. Despite the development of novel clinical strategies, their applicability in terms of duration is limited with the acute stage [ 1]. Thrombolytic tissue plasminogen activator (tPA) treatment could be administered within 4.5 h from stroke onset [2]. In addition, endovascular thrombectomy procedure, an innovative strategy for mechanical removal of the clot, is performed for patients not eligible for tPA administration [3]. The need for prompt action in stroke patients is necessary to provide regular circulation in the brain and to reduce the ischemia-dependent injury. Even the patients are lucky enough to retrieve the brain functions, long-lasting neurological effects may be obtained [4]. These limitations make the development of effective neuroprotective and neurorestorative substances favored. To further broaden the restrictions of this area, pharmacological, biotechnological, and electrophysiological therapies that enhance neuroplasticity are being investigated with a great interest [5].

Neurotrophic factors are one of the specific biomolecules that support neuroplasticity and neurogenesis. Hereby, as they hold great potential for repair of neurodegeneration, characteristics of neurotrophic factors were examined in many aspects [6]. Glial cell-line derived neurotrophic factor (GDNF) is one of the most well studied neurotrophic factor due to its role on dopaminergic neurons by inducing dopamine (DA) release in the striatum and promoting morphological differentiation of striatal neurons [7–9]. The accumulating evidence suggests that GDNF can regulate striatal neurons’ survival, migration, and differentiation [10]. Previous studies have demonstrated that there is a close interaction or crosstalk between GDNF and nuclear receptor related 1 (Nurr1), an essential orphan ligand-activated transcription factor directly related with dopaminergic functions. Nurr1 is highly expressed in the developing and ventral midbrain and regulates the DA metabolism and transportation. It was speculated that blockade of GDNF in nigral DA neurons decreased the Nurr1 and downstream target GDNF receptor tyrosine kinase (RET) expression [11, 12 ].

Dopaminergic neurons are the primary source of DA involved in motor and reward-related brain functions [13]. In addition to the midbrain tegmentum, there is a small portion of dopaminergic neurons located in the striatum. Striatal dopaminergic neurons are unique subtypes of gamma-aminobutyric acid (GABA)-ergic interneurons, which are able to produce both DA and GABA neurotransmitters [14]. Even though the exact role of striatal dopaminergic neurons is still a mystery, these locally acting neuronal subgroups are known to be increased in number in animal models of Parkinson’s disease (PD) [15]. DA also plays an essential role in other neurodegenerative disorders like Huntington disease (HD) and multiple sclerosis (MS) [16]. Recent clinical and experimental findings demonstrated that alterations in DA balance play a pivotal role in the motor and cognitive symptoms of HD and decreased behavioral flexibility [17]. In addition, it is speculated that dopaminergic signaling pathway regulates both de-myelination and re-myelination in MS [18]. Considering this response by dopaminergic striatal neurons, there must be a compensatory mechanism carried out for dealing with the neuronal degeneration [19].

Post-ischemic stages accompany enormous physiological events; transient occlusion of blood flow gives rise to sudden cell death and initiates several molecular processes in the primarily affected core region [ 20]. We previously demonstrated the potential role of lentiviral GDNF (Lv-GDNF) delivery on long term neurological recovery [10]. Herein, our scope was investigating the possible impact of GDNF treatment on striatal dopaminergic neurons in the acute phase of stroke. In this context, we hypothesized that GDNF could be used as a neuroprotective agent for maintaining dopaminergic neurons’ functions and, in the meanwhile, we aimed to uncover the mechanisms behind GDNF’s action.

## 2. Materials and methods

### 2.1. Ethics statement

Experiments were performed in accordance to National Institutes of Health (NIH) guidelines for the care and use of laboratory animals and approved by local government authorities (İstanbul Medipol University, Animal Research Ethics Committee). All animals were held in a daily lighting period of 12 h of light and 12 h of darkness. Investigators were blinded for experimental groups at all stages of experiments and data analysis.

### 2.2. Experimental groups

Experiments were performed using 14 male C57BL/6J mice (8–10 weeks, 20–25 g). Adult mice were randomly divided into two groups and administered with intrastriatal delivery of vehicle (Lv-GFP) or lentiviral vector expressing GDNF (Lv-GDNF). Ten days after intrastriatal injection, mice were subjected to 30 min of middle cerebral artery occlusion induced focal cerebral ischemia followed by 72 h reperfusion. Seventy-two hours after ischemia, mice were deeply anesthetized, decapitated, and brains were frozen with dry ice. Following, brains were sectioned into 18 μm coronal portions using a cryostat (CM1850-UV; Leica, Germany).

### 2.3. Lentivirus production

A second-generation lentivirus packaging system was used to produce vectors according to safety protocols. After total RNA extraction from SH-SY5Y cell cultures (80004, Qiagen, Germany), complementary DNA (cDNA) was reversed from RNA templates (04896866001, Roche). Human GDNF transcript variant 1 (NCBI Reference Sequence: NM_000514.3) was amplified using the defined primers (Forward: 5’- AGT CAG GTA CCA TGA AGT TAT GGG ATG TCG TGG -3’ and reverse 5’- AGT CAG CGG CCG CGG AGT CAG ATA CAT CCA CAC C -3’). Both PCR products and lentiviral expression plasmid pLenti (LV590 Applied Biological Materials, Canada) were fast digested with restriction enzymes KpnI (FD0524, Thermo Fisher Scientific, USA) and NotI (FD0593, Thermo Fisher Scientific, USA). Next, ligation reaction was performed with T4 DNA ligase (EL0014, Thermo Fisher Scientific, USA) for obtaining an expression plasmid with GDNF insert. pMD2.G and psPAX packaging plasmids were kindly provided by Dr. Didier Trono (Ecole Polytechnique Federale De Lausanne, France). The plasmids were used as complementary vectors for packaging the lenti-viral system. HEK293T cells (6 x10^6^ cells) were seeded on 10 cm plates. Transfection process was carried out using Lipofectamine 3000 (L3000015, Thermo Fisher Scientific, USA) for generating DNA-lipid complex. pLenti (7 μg), pMD2.G (3.5 μg), and psPAX (7 μg) vectors mixed in lipid complex was applied to cells drop by drop. The medium was supplemented with fresh DMEM 6 h after transfection, and the cells were incubated at 37 °C in a moist environment containing 5% CO_2_. The whole medium was harvested twenty-four and fifty-two hours after transfection, centrifuged for ten minutes at 2000 rpm, and screened with a low binding filter with a pore size of 0.45 μm. Virus particles were collected after ultracentrifugation at 100,000 g for 2 h and dissolved in Dulbecco’s Phosphate-Buffered Saline (DPBS; P04-3650, Pan Biotech, Germany) without calcium and magnesium. As a control, an expression plasmid without any insert DNA was packed using the same protocol as the GDNF-containing one.

### 2.4. Calculation of virus titer

HEK293T cells were exposed to ten-fold serially diluted virus particles ranging from 10^–1^ to 10^–4^, and the medium was replaced every day for three days. Cells were treated with trypsin, inactivated with culture media, spun, and resuspended in cold phosphate buffer saline (PBS) for fluorescence activated cell sorting (FACS) study after adequate GFP signaling (Becton Dickson Influx cell sorter, USA). Wells containing cells expressing between 1%–20% GFP were used to evaluate the titer of the virus particles. According to the formula “Virus titer (*TU/ml=FxCn/(V(ml)*) x Df “ multiplicity of infection (MOI) was calculated as 10^8^. F represents the frequency of GFP-expressing cells, *Cn* represents the total number of cells infected (4x10^5^), *V* represents the volume of the inoculum (1 mL), and *Df* represents the virus dilution factor in this calculation. According to titer measurements, viral vectors were used at a concentration of 10^8^ particles in 2 μL PBS [21].

### 2.5. Virus injection

Mice were anesthetized with 1% isoflurane and placed on a stereotactic frame (Stoelting, Illinois, USA). After drilling the skull according to the unilateral coordinate corresponding to the striatum (AP: bregma stage, ML: −2.5 mm, DV: +3 mm) Lv-GDNF or Lv-GFP viral particles were administered into the left hemisphere via micro-syringe pump controllers (Micro 4; World Precision Instrument, USA).

### 2.6. Induction of cerebral ischemia

Mice were anesthetized with isoflurane (1%) and 30% O_2_, reminder N_2_O. A feedback-controlled heating system was used to maintain the necessary rectal temperature of 36.5–37.0 °C (MAY instruments, Ankara, Turkey). Laser Doppler Flowmetry measured cerebral blood flow (CBF) during MCAO and reperfusion (LDF). To do so, tissue adhesive was used to bind a flexible 0.5 mm fiber optic probe (Perimed, Sweden) to the intact skull above the MCA territory (AP: +2 mm and ML: +6 mm from the bregma). An intraluminal filament technique was used to establish focal cerebral ischemia [22, 23 ]. The left common and external carotid arteries were separated and ligated after a small midline neck incision. Microvascular clips were used to temporarily ligate the internal carotid artery (FE691; Aesculap, Germany). For MCAO, a 180–190 μm silicon coated (Xantropen; Bayer Dental, Japan) 8.0 nylon monofilament (Ethilon; Ethicon, Germany) was implanted into the normal carotid artery via a small incision and advanced 9 mm distal to the carotid bifurcation. Reperfusion was started 30 min later when the filament was removed. LDF recordings were continued for 20 min to check reperfusion. Wounds were gently sutured after surgery, anesthesia was ended, and mice were returned to their cages.

### 2.7. Immunofluorescence

For immunofluorescence analysis of tyrosine hydroxylase (TH), sections from the bregma level were fixed in 4% paraformaldehyde (PFA), rinsed, and submerged in 0.1 M phosphate-buffered saline (PBS) containing 0.3% Triton X-100 (PBS-T) and 10% normal goat serum (NGS) for 1 h before being pretreated with citrate buffer for antigen retrieval. The sections were then incubated overnight at 4 °C with antibodies against TH (AB152, Millipore, USA). The next day brain sections were incubated with an Alexa Fluor 488 conjugated goat anti-rabbit secondary antibody (A11034, Thermo Fisher Scientific, USA) at room temperature. Then, sections were examined under confocal microscopy (LSM 780, Carl Zeiss, Germany) after counterstaining with 4′,6-diamidino-2-phenylindole (DAPI; D9542, Sigma Aldrich, USA)) for detecting the nuclei. Positively stained cells from 9 distinct regions of interest (ROI), each having 62,500 μm^2^ area, were analyzed from the ischemic striatum. For validating GNDF expression, sections from the bregma level were stained with anti-GDNF primary antibody (sc-13147, Santa Cruz Biotechnology, USA) and Alexa Fluor 488 conjugated goat anti-mouse secondary antibody (A11001, Thermo Fisher Scientific) by following the same protocol.

### 2.8. Fluoro Jade C staining

To analyze neuronal degeneration after focal cerebral ischemia, Fluoro Jade C staining was performed. Coronal sections were fixed in PFA and washed with 0.1 M PBS and dH_2_O successively. Next, the sections were incubated in 80% ethanol with 1% NaOH for 5 min. After rinsing in 70% ethanol for 2 min, the sections were washed in dH_2_O and incubated in 0.06% KMnO_4_ solution for 20 min. Then, the slides were rewashed in dH_2_O and treated with Fluoro Jade C working solution (A6325, Millipore) for 10 min in the dark. After several washing steps, the brain sections were counterstained with DAPI for nuclear signal. Finally, they were air dried, immersed with xylene, and mounted with entellan (1079600500, Sigma Aldrich). Degenerated neurons were monitorized and evaluated with confocal microscopy (LSM 780, Carl Zeiss). Cells from 9 distinct ROI, each having 62,500 μm^2^ area, were analyzed from the ischemic striatum.

### 2.9. Western blot analysis

Ischemic and non-ischemic striatal regions were isolated from the brain tissue samples. Radioimmunoprecipitation assay (RIPA) lysis buffer (89900, Thermo Fisher Scientific) containing a protease and phosphatase inhibitor mixture (5872, Cell Signaling Technology, USA) was used to homogenize samples from the same groups. After 15 min of centrifugation at 14,000 rpm, proteins were removed. BCA protein assay kit (23227; Thermo Fisher Scientific) was used to determine protein concentrations. Just after concentration determination, an equivalent volume of protein samples (20 μg) was loaded into 4%–20% TGX (Tris-glycine) gels (4561094, Biorad Life Sciences, USA) run for 1 h at 150 V and then transferred to PVDF membranes (162-0174, Biorad Life Sciences, USA). Membranes were blocked for 1 h at room temperature with 5% non-fat dry milk dissolved in Tris-buffered saline containing Tween-20 (TBS-T). Antibodies against GDNF (sc-13147, Santa Cruz Biotechnology), Nurr1 (sc376984, Santa Cruz Biotechnology), Gap43 (5307, Cell Signaling), iNOS (sc-650, Santa Cruz Biotechnology), nNOS (ab76067, Abcam, United Kingdom), NF-κB (ab16502, Abcam) and tyrosine hydroxylase (AB152, Millipore) were diluted in %5 non-fat dry milk (sc-2324, Santa Cruz Biotechnology) with TBS-T and incubated overnight on membranes. Next day after several washing with TBS-T membranes were incubated with the suitable secondary antibodies goat anti-rabbit-HRP (7074, Cell Signaling Technology) or goat anti-mouse-HRP (7076, Cell Signaling Technology) for 1 h at room temperature. Then, TBS-T washed blots were improved with a chemiluminescent substrate (ECL) (K-12043-D10; Western Bright Sirius, Advansta, USA) and visualized using a CCD camera cabinet (Fusion FX7, Vilber, Germany). Membranes were stripped and re-probed with an anti-actin (4970, Cell Signaling) antibody for protein normalization. Image J program (National Institute of Health, USA) was used to measure the densitometrical levels of proteins.

### 2.10. Statistical analysis

A software package was used to analyze statistical results (SPSS for Windows; SPSS Inc., Chicago, IL, USA). The independent-sample t test was used to determine the variations between the classes, followed by least significant differences (LSD) test. All values were expressed as mean ± standard deviation. The p values of less than 0.05 were considered significant.

## 3. Results

### 3.1. GDNF overexpression was validated by Western blot and immunofluorescence

**Figure 1:**
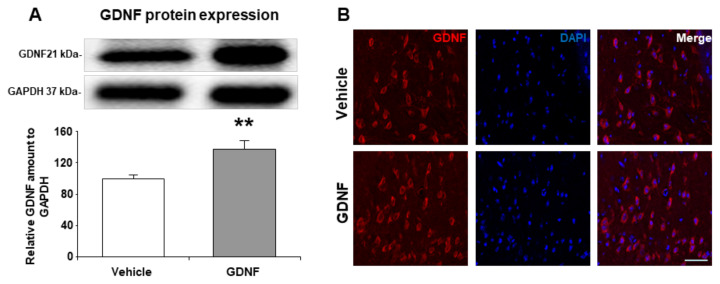
Validation of GDNF overexpression. GDNF overexpression was confirmed via (A) Western blot and (B) immunofluorescence staining from the striatum level. A representative image of Western blot analysis from three independent experiments was given above their corresponding graph. Data are represented as mean ± standard deviation values of three independent experiments. **p < 0.01 compared to vehicle treated group. The scale bar represents 40 μm.

Striatal tissue samples from the injection area were used to confirm exacerbation in the amount of GDNF protein expression. Results demonstrated that GDNF protein content was significantly upregulated upon lentivirus treatment (p = 0.004) (Figure 1A). In addition to this, immunofluorescence staining of GDNF supported this finding (Figure 1B).

### 3.2. Neither TH positively stained neurons nor TH expression in the ischemic striatum was different from control animals

**Figure 2:**
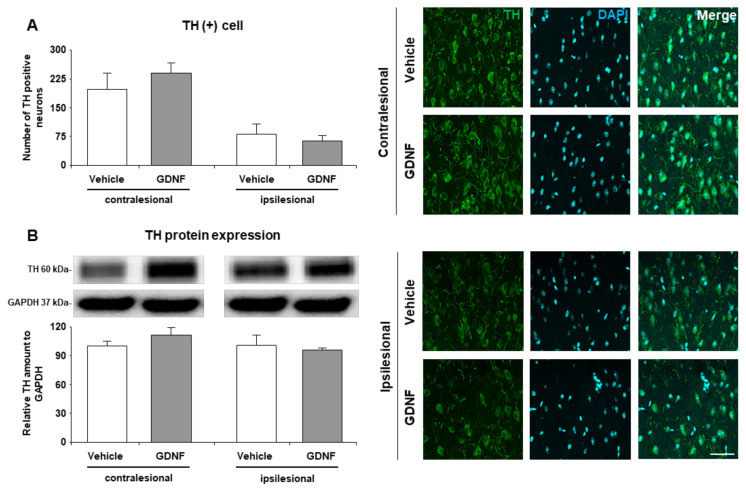
Analysis of striatal dopaminergic neurons. (A) The number of TH positive neurons was assessed from the immunofluorescence staining from the striatum. In addition, (B) TH protein expression was analyzed from both ipsilesional and contralesional striatum. A representative image of Western blot analysis from three independent experiments was given above their corresponding graphs. Data are represented as mean ± standard deviation values of three independent experiments. The scale bar represents 40 μm.

Results showed that the number of TH positive neurons reduced due to ischemic injury (Figure 2A). However, there was no significant difference between the vehicle or GDNF treated groups for the numbers of striatal dopaminergic neurons both in the ipsilesional (p = 0.593) and contralesional hemispheres (p = 0.425). Also, TH protein expression analysis indicated that TH protein expressions were similar between the vehicle and GDNF treated animals for both sides independent of the treatment (Figure 2B).

### 3.3. GDNF overexpression in the ischemic striatum reduced neuronal degeneration

**Figure 3:**
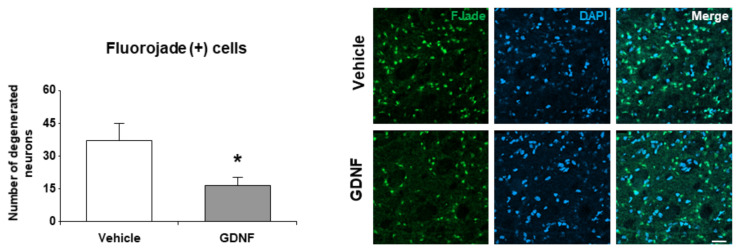
Analysis of degenerating neurons by Fluoro Jade C staining from the striatum level. Data are represented as mean ± standard deviation values of three independent experiments. *p < 0.05 compared to vehicle treated group. The scale bar represents 40 μm.

The number of degenerated neurons after focal cerebral ischemia was analyzed using Fluoro Jade C staining, which is generally used to detect degenerated neurons (Figure 3). Results demonstrated that the number of degenerated neurons in the ischemic striatum was significantly reduced in the GDNF treated group (p = 0.045).

### 3.4. GDNF delivery regulated the production of the orphan nuclear receptor Nurr1 and axonal membrane protein GAP43

**Figure 4:**
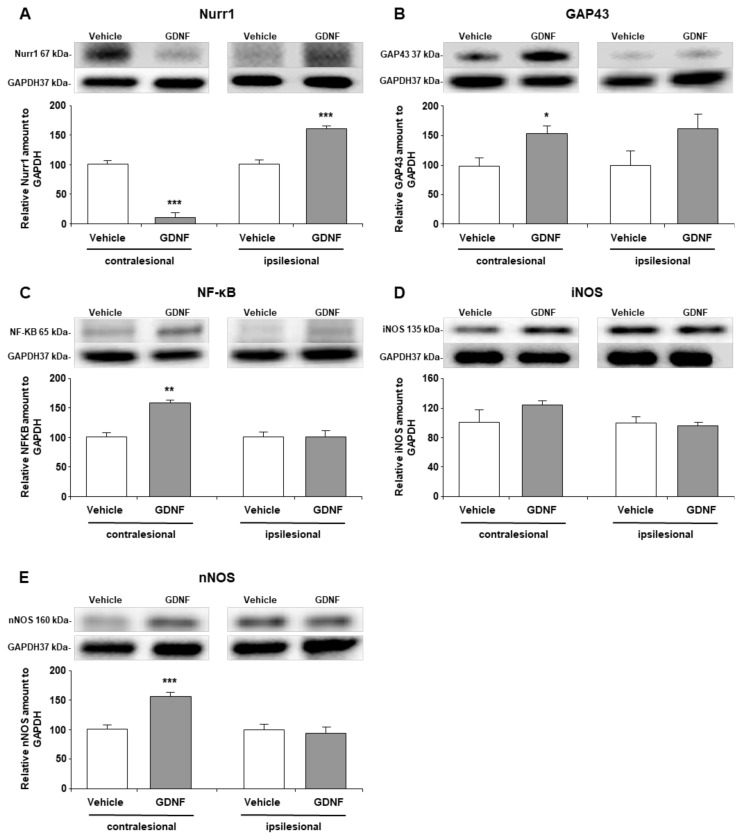
Western blot analysis of (A) Nurr1, (B) GAP43, (C) iNOS, (D) iNOS, and (E) nNOS proteins. Representative images of Western blot analysis from three independent experiments were given above their corresponding graph. Data are represented as mean ± standard deviation values of three independent experiments. ***p ≤ 0.001/ **p <0.01/ *p < 0.01 compared to vehicle treated group.

Western blot analysis demonstrated that lentiviral GDNF administration significantly increased the orphan transcription factor nuclear receptor-related 1 protein (Nurr1) expression in the ischemic striatum (p ≤ 0.001) (Figure 4A). However, Nurr1 protein expression was significantly reduced in the contralesional striatum (p = 0.001). On the other hand, GDNF treatment slightly but not significantly (p = 0.056) increased growth associated protein 43’s (GAP43) protein expression, which plays essential role after neuronal injury in the ipsilesional striatum (Figure 4B). Notably, GAP43 was upregulated in the Lv-GDNF applied animals in the contralesional striatum (p = 0.041).

### 3.5. GDNF overexpression regulated proteins related to inflammatory response and synaptic function

Expression of nuclear factor kappa B (NF-κB), a well-known regulatory transcription factor of inflammatory processes, and major enzymes responsible for the production of vasodilator nitric oxide (NO), inducible nitric oxide synthase (iNOS), and neuronal nitric oxide synthase (nNOS) were analyzed by Western blot. The data indicated that NF-κB protein expressions increased significantly (p = 0.001) with Lv-GDNF in the contralesional hemisphere (Figure 4C). At the same time, there was only a moderate rise (p = 0.083) for inducible nitric oxide synthase (iNOS) (Figure 4D). Like NF-κB, nNOS protein expression increased significantly (p ≤ 0.005) with Lv-GDNF in the contralesional hemisphere (Figure 4E).

## 4. Discussion

This study addressed the fundamental question of the possible role of lentivirally induced GDNF expression on dopaminergic neurons in the case of acute neuronal degeneration. For this purpose, we designed an experimental procedure firstly by focusing on the survival of tyrosine hydroxylase (TH) positive dopaminergic neurons.

Previous studies demonstrated that GDNF improves behavioral functions, promotes neurogenesis, reduces infarct size, increases synaptic plasticity, and decreases apoptosis when applied as a recombinant protein, a TAT-fusion protein or via a viral vector in the post-acute phases of stroke [ 10, 21, 24 ]. In this way, GDNF provides long-term neurological recovery by remodeling the brain. Considering the limited time window for therapeutic applications in clinics, the acute phase of stroke becomes more of an issue. Therefore, we chose 30 min of intraluminal MCAO followed by 72 h reperfusion model, which is the more appropriate model to evaluate disseminated neuronal injury in the acute phase of stroke [23]. While beneficial effects of GDNF on neuronal functions have been reported in most previous studies, our data will contribute to the literature with the perspective of dopaminergic functions. Since the accumulating evidence also suggests that GDNF signaling in the striatum is essential for neuromodulation of dopaminergic neurons, it requires constant stimulation [9].

TH is a -limiting enzyme taking place during the conversion of tyrosine to DOPA and is generally considered a dopaminergic neuron marker [25]. Usually, dopaminergic neurons have a crucial role in controlling numerous brain activities such as motor and cognitive functions, including coordination of voluntary movements, mood, reward, and stress-related responses [26]. It is known by the past works that striatum harbors a small number of dopaminergic neurons whose number increase with pathological conditions [15]. At this point, striatal dopaminergic neurons may be a great of interest in neurodegeneration to develop novel therapeutic strategies.

After verifying the significant upregulation in GDNF levels, TH expressing neurons were evaluated for finding the possible impact of GDNF. It is evident that, Lv-GDNF delivery didn’t have a profound action on dopaminergic neuron survival as expected. This may be due to the severity of the pathophysiological events in the acute stroke such as intense necrosis, inflammation energy failure and excitotoxicity [ 27]. This catastrophic environment doesn’t exactly provide representation of recovery related processes. For this reason, overall consequences of GDNF therapy on dopamine system can be investigated in long term. As noted earlier, it is well-known with numerous experiments that GDNF has such a power to induce neuroprotection on dopaminergic neurons under different pathological conditions [28].

Fluoro Jade C is an efficient fluorescent dye used as a degenerating neuron marker by many researchers. Positive labeling by Fluoro Jade C indicates the existence of degenerating neurons independent of the way of cell death mechanism. Necrotic, apoptotic, and autophagic cells can be detected in this manner easily [29]. Glutamate excitotoxicity related neurotoxicity, a hallmark of ischemic stroke pathophysiology can also be screened through Fluoro Jade staining [30]. According to our analysis, Lv-GDNF delivery protected neuronal degeneration. Although GDNF has no apparent effect on dopaminergic neurons, it can modulate dopaminergic neurons in the aspect of neuronal death rather survival.

For further analyzing the molecular basis of dopaminergic regulation Nurr1, a significant nuclear receptor directly related with dopaminergic functions was evaluated. Besides being an extraneous marker for dopaminergic neurons, Nurr1 also targets some regulatory elements, which designates GDNF signaling [11]. In this way, Nurr1 and GDNF have a reciprocal interaction. Nurr1 expression was demonstrated to be associated with dopamine (DA) neurotransmission, and, by doing so, it increases TH expression in dopaminergic neurons [31]. In this perspective, the dramatic rise in the expression of Nurr1 in the ipsilateral striatum could be a straight consequence of GDNF stimulation. According to our data, contralesional striatum displayed an opposite outcome in terms of Nurr1 expression.

An ischemic pathology induces impairments in both hemispheres accordingly, neuronal remodeling is presumable as a bilateral activation during recovery [32]. It was an outstanding finding to show the relationship between functional recovery and contralesional plasticity through the reorganization of growth-associated proteins [10]. In this context, distinct patterns of Nurr1 expression in either sides may be due to establishing the interhemispheric balance. Despite having a similar trend of increment upon Lv-GDNF treatment, growth cone protein GAP43 was significantly higher only in the contralateral hemisphere. Previously, it was demonstrated that insufficient neurite regeneration associated loss of dopaminergic neurons correlates with reduced striatal expression of GAP43 [33].

Lastly, within the scope of this study, inflammation-related proteins were examined. There was a similar pattern of expression for proteins NF-κB, iNOS, and nNOS whose amounts increased in the contralateral hemisphere. This may be due to decreased levels of Nurr1, since it is known that Nurr1 is a potent antiinflammatory factor, which can modulate inflammatory responses [34]. NF-κB is a versatile transcription factor involved in multiple cellular functions in the nervous system. Due to its master regulatory role for inflammation, it is also quite critical for the regulation of apoptosis [35]. Here, when NF-κB is considered as a junction between inflammation and apoptosis pathways, there may be possible crosstalk between them. One of the downstream pro-inflammatory mediators of NF-κB is iNOS whose activation is expected to contribute to ischemic damage [35]. According to the basic knowledge about inflammatory processes, nNOS and iNOS are direct supporters of tissue destruction through high-output NO (nitric oxide) synthesis. However, recent data reveals their protective role by virtue of regulatory functions [36, 37 ]. Even though nNOS and iNOS seem to play a destructive role, one such protective mechanism can be maintained over NO production, which contributes to post-ischemic recovery by stimulating vasodilation and increasing blood flow [38]. These results elucidate that those inflammatory mechanisms can be regulated in a complicated manner, and GDNF has a part in this organization by dynamically modulating contralesional molecular processes.

## 5. Conclusion

In recent years, neurotrophic factors such as nerve growth factor, brain-derived neurotrophic factor, and neurotrophin have been used to treat neurodegenerative diseases, including cerebral ischemia. The approach presented here was constructed on the behavior of striatal dopaminergic neurons after GDNF treatment for ischemic damage. The accumulating evidence indicated the potential curative role of GDNF on dopaminergic neurons. Even though our previous study showed the increased neuroplasticity, neurogenesis, and neuronal re-modeling upon Lv-GDNF delivery, the current study showed the relationship between GDNF and survival of the striatal dopaminergic neurons. However, this study highlighted the importance of Nurr1 as a major regulatory element and its possible interaction with GDNF. It is interesting to note that finding novel therapeutic strategies for ischemic stroke will be available only with better understanding and explaining the ischemic pathology with different aspects. In this context, GDNF and dopaminergic neurons perfectly match puzzle pieces to enlighten ischemia pathophysiology and open ways for regenerative treatments.
